# Effect of Heat Treatment on Microstructure and Mechanical Behavior of Ultrafine-Grained Ti-2Fe-0.1B

**DOI:** 10.3390/ma16082955

**Published:** 2023-04-07

**Authors:** Yaoyao Mi, Yanhuai Wang, Yu Wang, Yuecheng Dong, Hui Chang, I. V. Alexandrov

**Affiliations:** 1 College of Materials Science and Engineering/Tech Institute for Advanced Materials, Nanjing Tech University, Nanjing 211816, China; myy20177030139@163.com (Y.M.); wangyu202203@163.com (Y.W.); ch2006@njtech.edu.cn (H.C.); 2 Casting and Forging Branch, Lanzhou LS Group Co., Ltd., Lanzhou 730314, China; wangchu36@163.com; 3 Department of Materials Science and Physics of Metals, Ufa University of Science and Technology, Ufa 450076, Russia

**Keywords:** thermal stability, ultrafine-grained titanium alloy, equal channel angular pressing, Fe segregation, TiB phase

## Abstract

In the present study, a novel Ti-2Fe-0.1B alloy was processed using equal channel angular pressing (ECAP) via route Bc for four passes. The isochronal annealing of the ultrafine-grained (UFG) Ti-2Fe-0.1B alloy was conducted at various temperatures between 150 and 750 °C with holding times of 60 min. The isothermal annealing was performed at 350–750 °C with different holding times (15 min–150 min). The results indicated that no obvious changes in the microhardness of the UFG Ti-2Fe-0.1B alloy are observed when the annealing temperature (AT) is up to 450 °C. Compared to the UFG state, it was found that excellent strength (~768 MPa) and ductility (~16%) matching can be achieved for the UFG Ti-2Fe-0.1B alloy when annealed at 450 °C. The microstructure of the UFG Ti-2Fe-0.1B alloy before and after the various annealing treatments was characterized using electron backscatter diffraction (EBSD) and transmission electron microscopy (TEM). It was found that the average grain size remained at an ultrafine level (0.91–1.03 μm) when the annealing temperature was below 450 °C. The good thermal stability of the UFG Ti-2Fe-0.1B alloy could be ascribed to the pinning of the TiB needles and the segregation of the Fe solute atoms at the grain boundaries, which is of benefit for decreasing grain boundary energy and inhibiting the mobility of grain boundaries. For the UFG Ti-2Fe-0.1B alloy, a recrystallization activation energy with an average value of ~259.44 KJ/mol was analyzed using a differential scanning calorimeter (DSC). This is much higher than the lattice self-diffusion activation energy of pure titanium.

## 1. Introduction

The excellent physical and chemical properties of ultrafine-grained (UFG)/nanocrystalline (NC) materials fabricated using severe plastic deformation (SPD) techniques, such as a high strength, superplasticity, electroconductivity and corrosion resistance, have been widely studied for decades [1,2,3] to develop their application in dental implants and the compressor blades of gas turbine engines [4,5]. However, in view of the existence of their relatively large stored energy and non-equilibrium grain boundary characteristics, UFG/NS materials are generally considered unstable, as they can induce recrystallization and grain growth at elevated temperatures, leading to the loss of their superior properties. Therefore, ever more studies are concentrating on the thermal stability of UFG/NC materials and are achieving valuable results [6,7,8].

Two approaches are always adopted to improve the thermal stability of UFG/NC materials, i.e., reducing the grain boundary energy from the thermodynamical aspect and inhibiting the mobility of the grain boundaries from the kinetic aspect. For example, the thermal stability of low angle grain boundaries (LAGBs) [9] and coherent twin boundaries [10] is superior to that of high angle grain boundaries (HAGBs), as the energy stored at LAGBs and twin boundaries is much lower than at HAGBs. Moreover, the notable thermal stability of NC pure Cu and Ni metals below a critical grain size was found to be even higher than the recrystallization temperature of coarse grains, which was explained by the autonomous grain boundary evolution to low energy states due to the activation of partial dislocations in plastic deformation [11].

Generally speaking, alloying using specialized elements is one of the most effective methods for improving the thermal stability of UFG/NC materials, allowing a decrease in boundary energy via the segregation of the solute atoms at the grain boundaries, and postponing the mobility of the grain boundaries via the Zener pinning effect. This conclusion has been verified in several alloy classes, such as W-Ti, Cu-Ta and Fe-Zr [7,8,12]. Although few previous studies have concentrated on the thermal stability of the UFG/NC titanium alloy, titanium alloys are usually used at an elevated temperature due to their high temperature resistance [13,14,15].

Recently, accompanying the development of a low-cost titanium alloy, the mechanical behavior and phase transformation of binary Ti-Fe titanium alloys were studied intensively [16,17,18,19]. Our group found that the Ti-2Fe titanium alloy possesses improved mechanical properties, corrosion resistance and in vitro response, which shows its promise as a dental implant [20]. Furthermore, B microalloying is famous for its use in titanium alloys to improve the ingot structure, which is of benefit for the consequential forging and rolling process [21]. Hence, a novel Ti-2Fe-0.1B titanium alloy was designed by our team, developed and additively manufactured using several advanced technologies, such as severe plastic deformation and a thermal hydrogenated process [22,23,24]. As we all know, the B element addition in titanium alloys could benefit their thermal stability because of the TiB needles present at the grain boundaries, which restrict their mobility at high temperatures due to the Zener drag mechanism. On the other hand, G. Lütjering also showed how Fe has an inhibitory effect on the grain growth of titanium alloys at the micron grain scale after comparing two different Fe levels (0.15% and 0.03%) [25]; however, the study of the Fe alloying effect on the thermal stability of a UFG/NC titanium alloy is rarely reported. Considering that post-annealing is a common method used to optimize the microstructure and mechanical behavior of deformed and welded materials [26,27,28,29,30], as well as to fix the issue of thermal stability of the UFG/NC materials mentioned above, it is interesting to study the effect of heat treatment on the microstructure and mechanical behavior of the UFG/NC Ti-2Fe-0.1B alloy.

## 2. Materials and Experimental Procedures

### 2.1. Materials

A novel Ti-2Fe-0.1B alloy (hereinafter referred to as TiFeB) was chosen for study in the present work. The actual chemical compositions (wt.%) are as follows: Fe, 1.89%; B, 0.08%; C, 0.014%; H, 0.0012%; O, 0.062%; N, 0.004%; Ti, balance.

**Figure 1:**
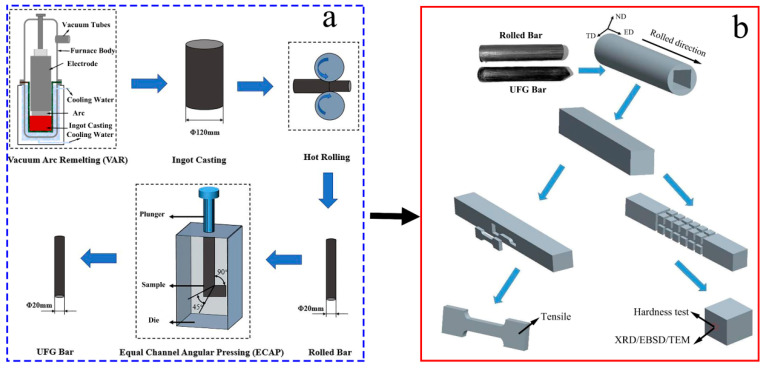
Schematic illustration of (**a**) the processing routes and (**b**) sample-selected plane for tests.

Figure 1a shows the schematic of the processing routes, which includes two steps. The ingot casting was produced using vacuum arc remelting (VAR). After that, via the processes of blank opening, precision forging and skin grinding, the TiFeB alloy bar was directly hot-rolled to a diameter of 20 mm. A glass protective lubricant was applied to reduce pollution during the rolling process. Then, the equal channel angular pressing (ECAP) method with an intersecting channel angle of 90° and an outer-arc angle of 45° was performed to refine the microstructure via route B_c_ (the billet is rotated 90° clockwise) [31] for 4 passes at 500 °C. The samples used for the microstructure observation and mechanical behavior tests were taken from the longitudinal section of the alloy bar; the specific sampling schematic diagram is shown in Figure 1b.

To investigate the influence of the annealing temperature (AT) on the mechanical properties and microstructures of the UFG TiFeB alloy, the isochronal and isothermal treatments were performed. Isochronal annealing was conducted at various temperatures from 150 °C to 750 °C with holding for one hour, and isothermal annealing was performed at temperatures from 350 °C to 750 °C with different holding times (15 min–150 min). Air cooling was used after annealing.

### 2.2. Microstructure Characterization

Electron backscatter diffraction (EBSD) and transmission electron microscopy (TEM) were used to characterize the microstructural evolution of the UFG TiFeB alloy and consequent annealed states. The EBSD samples were prepared by electro-polishing in a solution of 5% perchlorate acid and 95% ethanol at a temperature of 0 °C for 90 s with an applied potential of 25 V. The EBSD test was carried out on a Field Emission Scanning Electron Microscope (SEM, JSM-6700F) equipped with an Oxford Instruments Nano Analysis EBSD detector working at an accelerating voltage of 20 KV with a step size of 0.1 μm and a total scan area of 1600 μm^2^. The EBSD data was analyzed by an HKL Technology Channel 5 system. For the TEM investigation, the samples were subjected to mechanical grinding to a thickness of ~60 μm and then perforated using jet-polishing with an RL-I electrolytic twin-jet in a solution containing 6% perchloric acid, 34% butanol and 60% methanol with an accelerating voltage of 20 KV at temperature ranges of −15 °C to 0 °C. Finally, the TEM tests were performed on a FEI Talos F200X transmission electron microscope with an accelerating voltage of 200 kV.

### 2.3. Thermal Analysis

Thermal analysis was conducted on a Netzsch STA 449 F3 differential scanning calorimeter (DSC) instrument. The samples prepared for the DSC analysis were mechanically ground and polished with a mass of 15–25 mg, followed by sealing in Al pans and heating in a flowing Ar atmosphere from room temperature to 800 °C at constant heating rates of 5 °C/min, 15 °C/min and 25 °C/min. The DSC runs were repeatedly performed on each sample until the curve was smooth with a clear exothermic peak.

### 2.4. Mechanical Properties Tests

The hardness was tested using a HV-1000 Vickers hardness tester to evaluate the softening behavior during annealing with a load of 200 gf for 15 s. A total of 10 groups of data were measured with each group including 5 points. Finally, the average value of the 50 measurements was taken as the hardness value. The tensile samples with a gauge length of 10 mm, a width of 3 mm (gauge section) and a thickness of 2 mm used to characterize mechanical properties were annealed at 300 °C, 400 °C, 450 °C and 650 °C for 1 h. Tensile tests were carried out on a CSS-44100 electronic universal testing machine with a strain rate of 1 × 10^−3^ s^−1^ until the sample cracked, and the sample in each state was repeated at least three times. In addition, the ultimate tensile strength (*R_m_*), yield strength (*R_p0.2_*) and elongation to failure (*A*) were statistically calculated after each test.

## 3. Results

In order to analyze the thermal stability and microstructure evolution of the alloy, the mechanical properties of the samples under different annealing conditions were tested and the microstructure was characterized by EBSD and TEM. At the same time, the activation energy was determined based on the DSC results.

### 3.1. Initial Microstructure

**Figure 2:**
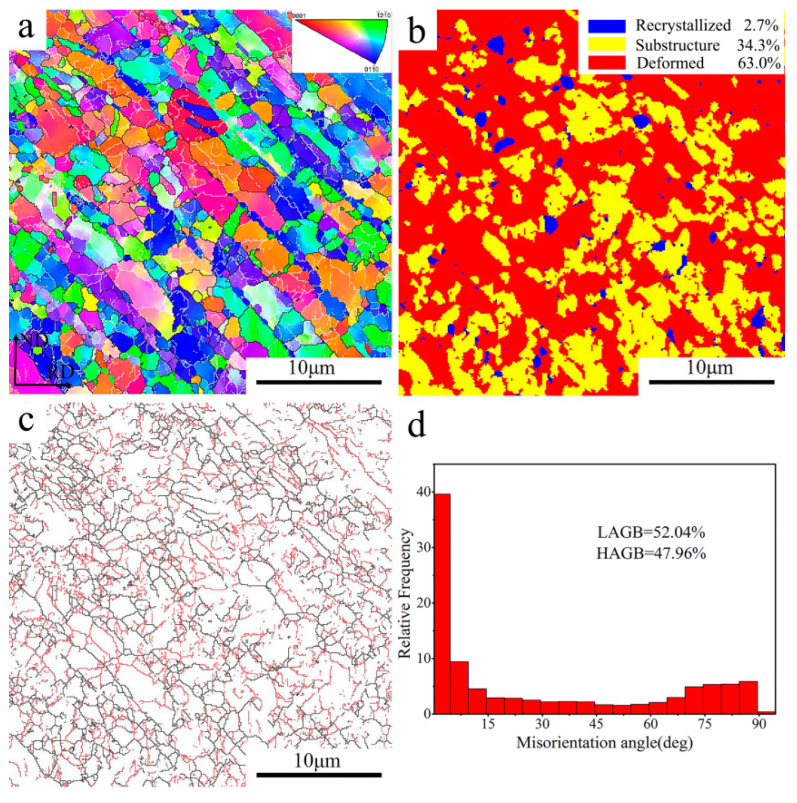
Microstructure characteristics of the UFG TiFeB alloy: (**a**) inverse pole figures (IPF) map; (**b**) recrystallization map; (**c**,**d**) maps of grain boundaries and the misorientation angle distributions.

The microstructure characteristic of the UFG TiFeB alloy is shown in Figure 2. The microstructure (Figure 2a) displays a remarkable grain refinement resulting from the large plastic deformation applied during ECAP. Compared to the original microstructure [15], the average grain size of the alloy decreased from 3.48 μm to about 0.24 μm after ECAP. According to the average misorientation angle of grains, the grains of this alloy can be divided into three types [32]: (i) average angle < 1°, recrystallized grains (represented in blue); (ii) 1° < average angle < 7.5°, substructure grains (marked in yellow); (iii) average angle > 7.5°, deformed grains (indicated in red). The recrystallization map characterized in Figure 2b shows that the microstructure of the UFG TiFeB alloy consists of deformed grains (~63%), substructure grains (~34.3%) and recrystallized grains (only ~2.7%). Due to the large shear strain during ECAP, serious grain fragmentation occurred and the number of sub-grains increased (Figure 2c,d), which resulted in the low angle grain boundaries of the UFG TiFeB alloy reaching 52.04%. In previous studies, Chen et al. [33,34] attributed the phenomenon of grain refinement during ECAP to continuous dynamic recrystallization (CDRX). In consideration of the high stacking fault energy in titanium alloy [35], Hoseini et al. [13] thought that the grain refinement starts from the formation of dislocation tangle zones close to the grain boundaries. In addition, during ECAP, the increase in strain and the dislocation density facilitate the coalescence of sub-grains, which contributes to the formation of LAGBs.

### 3.2. Mechanical Properties

**Figure 3:**
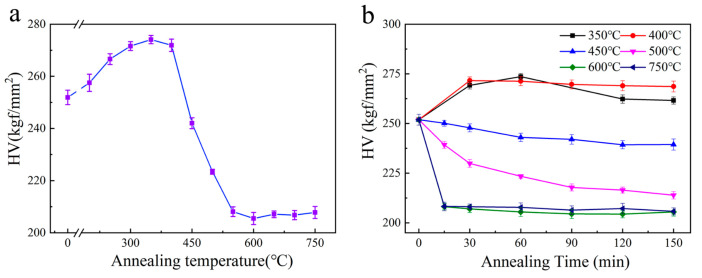
Microhardness development of the UFG TiFeB alloy: (**a**) isochronal annealing; (**b**) isothermal annealing.

Figure 3 shows the microhardness variation of the UFG TiFeB alloy with isochronal annealing at different temperatures for 60 min and isothermal annealing at 350–750 °C for 15–150 min. The microhardness of the UFG TiFeB alloy before annealing is approximately 252 HV. The microhardness variations are divided into three parts during isochronal annealing: (i) The microhardness increases up to 350 °C. A slight increase in microhardness approaching 277 HV is displayed when the AT increases to 350 °C. The hardening behavior above, induced by annealing with a low temperature and a short time, is a frequent phenomenon for UFG and NC materials during heat treatment [36,37,38,39], which can be ascribed to part of the non-equilibrium grain boundary restored before the grain growth of UFG and NC materials. (ii) The microhardness decreases with the AT increasing from 350 °C to 500 °C. Therein, annealed at 450 °C, the microhardness of this alloy sample is about 242 HV, which is almost the same level as in the as-received UFG state. (iii) The microhardness maintains stability after 550 °C.

To explain further, the microhardness of the UFG TiFeB alloy with isothermal annealing is shown in Figure 3b, which indicates that the microhardness variation exhibits two different stages: (i) 350 °C ≤ AT ≤ 400 °C. The microhardness increases at first, then reaches stability with the increase in holding time. (ii) AT = 450 °C. There is an initial slight decrease, then the microhardness is kept stable. (iii) 500 °C ≤ AT ≤ 700 °C. There is an initial rapid drop for the microhardness in a short time, then it is kept stable. Obviously, the microhardness of the UFG TiFeB alloy shows a consistent tendency to stabilize after an initial increase or decrease, which illustrates that microhardness is determined by the annealing temperature rather than the annealing time. In addition, it is worth noting that an almost constant value of microhardness is achieved for the sample when annealed at 450 °C for the whole holding time, which implies that the thermal stability of the UFG TiFeB alloy could hold at 450 °C even after long-term annealing.

**Figure 4:**
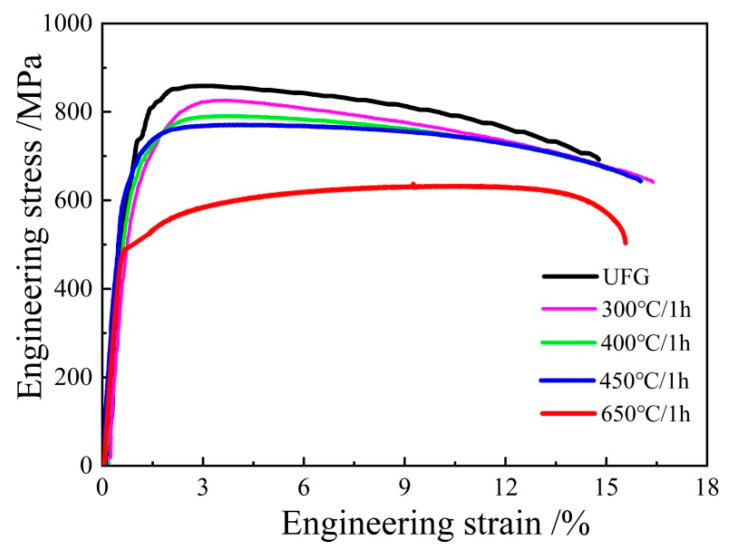
Engineering stress–strain curves of the UFG TiFeB alloy after isochronal annealing at different temperatures.

The mechanical properties of the UFG TiFeB alloy before and after isochronal annealing are shown in Figure 4. The ultimate tensile strength (UTS), yield strength (YS) and elongation to failure (A) of the UFG TiFeB alloy are 854 MPa, 637 MPa and 15%, respectively. With the increase in AT, all the strengths of the alloy show a continuous reduction. In particular, the UTS of the UFG TiFeB alloy decreases significantly to 635 MPa, and the elongation increases slightly to 17% after annealing at 650 °C. However, after annealing at 450 °C, the UFG TiFeB alloy still has a good combination of strength (UTS = 768 MPa, YS = 606 MPa) and elongation (A = 16%), which is superior to the mechanical properties of the Ti-6Al-4V alloy at a high temperature [40,41].

### 3.3. Microstructural Stability

**Figure 5:**
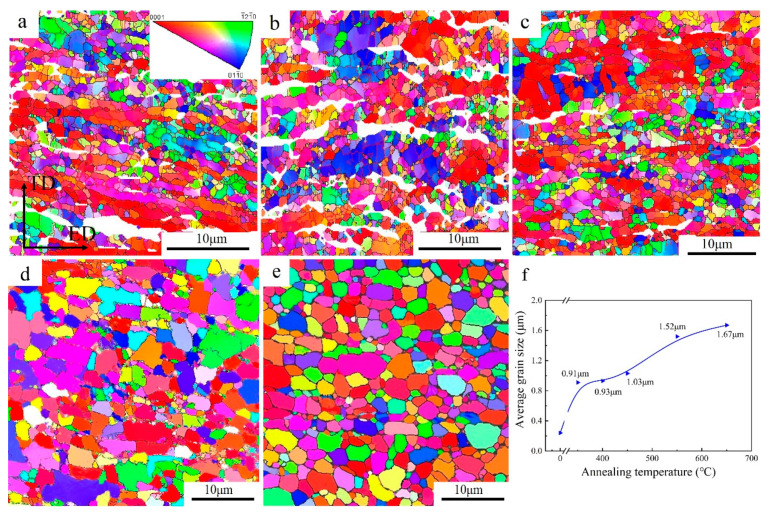
IPF maps and the variation of grain sizes in the UFG TiFeB alloy after annealing at (**a**) 350 °C, (**b**) 400 °C, (**c**) 450 °C, (**d**) 550 °C, and (**e**) 650 °C for 1 h. (**f**) The relationship of grain sizes vs. annealing temperatures.

In order to study the microstructure variation during recrystallization, the grain structures of the isochronous-annealed UFG TiFeB samples were characterized by EBSD. The results of the EBSD analysis, including IPF maps and the variation of grain sizes, are shown in Figure 5. Compared with the initial microstructure with an average grain size of 0.24 μm (Figure 2a), there is a slight change in average grain size (0.91–1.03 μm) when 300 °C < AT ≤ 450 °C. However, it is obvious that the average grain size increases to 1.52 μm with the AT increasing to 550 °C, and the grain morphology turns from elongated to approximately equiaxed grains. 

**Figure 6:**
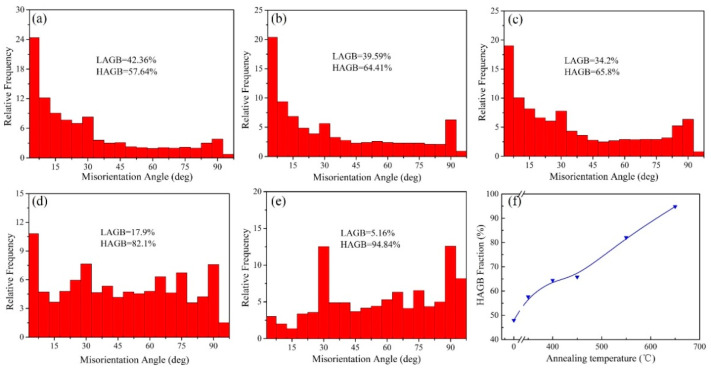
Misorientation angle distributions of the UFG TiFeB alloy after annealing for 1 h at different annealing temperatures of (**a**) 350 °C, (**b**) 400 °C, (**c**) 450 °C, (**d**) 550 °C, and (**e**) 650 °C. (**f**) The curve of HAGB variation with annealing temperature.

Figure 6 shows the misorientation angle distributions in the UFG TiFeB alloy after isochronal annealing at different annealing temperatures and the variation tendency of HAGBs for this alloy with annealing temperature. It is well known that the grain boundaries with a misorientation angle from 2° to 15° are defined as low angle grain boundaries (LAGBs) and the others (>15°) are regarded as high angle grain boundaries (HAGBs) [42]. Generally speaking, the fraction of HAGBs shows a tendency to increase with the annealing temperature. When the annealing temperature changes from 350 °C to 450 °C, the rate of increase for the HAGBs fraction is moderate, which is similar to the change of average grain size. For the sample with AT = 450 °C, the fraction of LAGBs decreases slightly to 34.2%, and the fraction of HAGBs increases to 65.8%. However, for the sample with AT = 550 °C, the fraction of LAGBs decreases sharply to 17.9%, while the fraction of HAGBs increases significantly to 82.1%.

**Figure 7:**
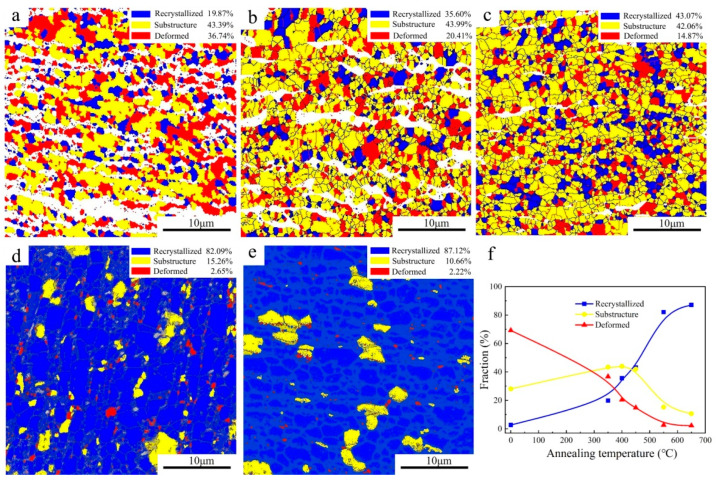
Recrystallization maps of the UFG TiFeB alloy after annealing for 1 h at different annealing temperatures of (**a**) 350 °C, (**b**) 400 °C, (**c**) 450 °C, (**d**) 550 °C, and (**e**) 650 °C. (**f**) The variation curve of the volume fraction with annealing temperature.

Figure 7 shows the recrystallization maps of the UFG TiFeB alloy at different annealing temperatures and the variation curve of the volume fraction of “recrystallization”, “substructure” and “deformed” in the “recrystallization” maps. Compared with the initial microstructure (Figure 2b), it is obvious that the change trend of the recrystallized grains fraction shows a slow increase (AT < 450 °C) and a rapid increase (AT > 450 °C), which is consistent with the HAGBs above. During the annealing process, the fraction of deformed grains and substructure grains decreases, while only the fraction of recrystallized grains increases, which indicates the replacement of the deformation microstructure by new recrystallized grains during annealing and the occurrence of static recrystallization (SRX) [43]. Therefore, the mechanical properties and microstructural stability are mainly determined by the SRX mechanism.

The increasing fraction of recrystallized grains, working as dislocation-free crystallites and accommodating more moving dislocations during plastic deformation [44], is responsible for the reduction in strength and the improvement in ductility (Figure 4) with the increase in the AT. Compared with the annealed samples, the fraction of LAGBs for the initial UFG TiFeB sample (Figure 2c,d) is the highest (52.04%), due to the production of abundant dislocations and sub-grains during the ECAP process. It was reported that the growth of recrystallized grains is driven by the previous strain process dislocations and the grain boundary stored energy [43]. During the process of recrystallization, the migration of the sub-grain boundaries with continuous absorption of dislocations makes LAGBs transform into HAGBs, resulting in the fraction of HAGBs increasing (Figure 6).

When AT = 450 °C, the fractions of recrystallized grains and sub-grains are almost identical (Figure 7c,f), achieving an optimal balance during SRX and showing obvious properties and microstructure stability. In addition, when AT= 650 °C, recrystallization is nearly complete, which makes the fraction of HAGBs increase significantly and strength decrease, corresponding to the migration of the grain boundaries during recrystallization.

## 4. Discussion

### 4.1. Dislocation Substructures

**Figure 8:**
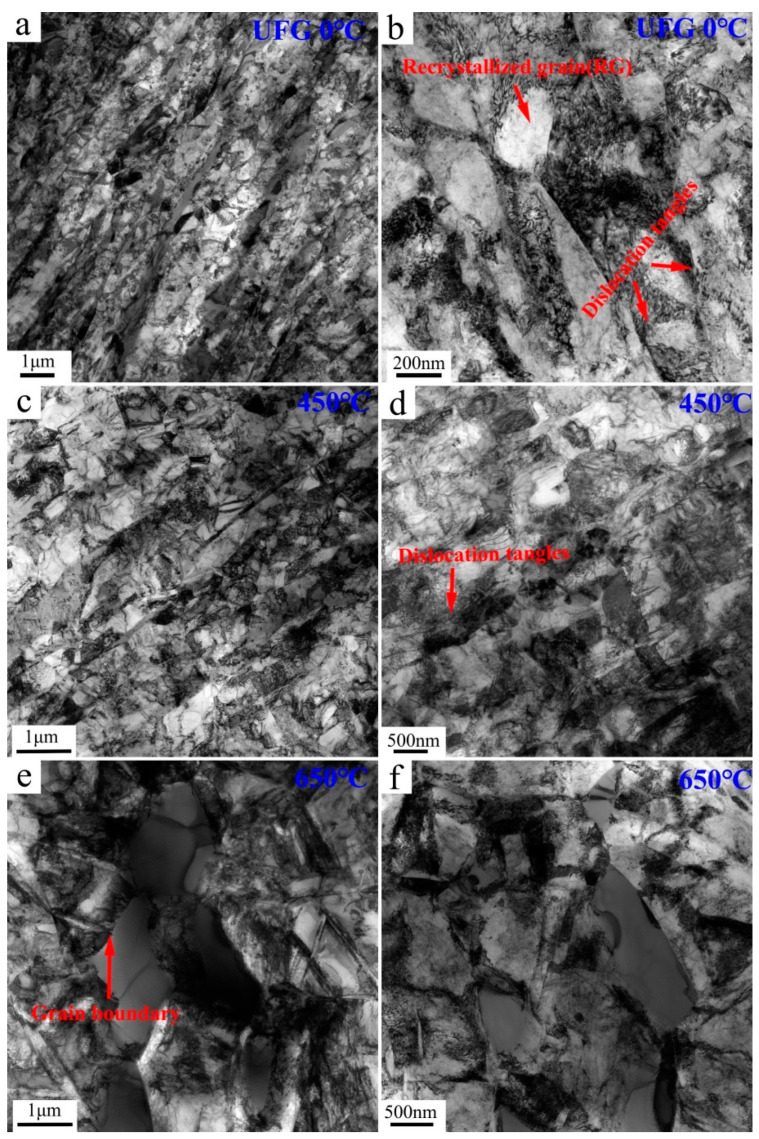
TEM micrographs of (**a**,**b**) the UFG TiFeB alloy samples, and the samples annealed at (**c**,**d**) 450 °C and (**e**,**f**) 650 °C.

The TEM micrographs of the UFG TiFeB alloy samples before annealing and after annealing at 450 °C and 650 °C are shown in Figure 8. Consistent with the results of EBSD above, the microstructure of the UFG TiFeB alloy consists of numerous sub-grains (Figure 8a). Recrystallized grains (RG), nearly free of dislocations, and the deformed grains with a larger number of dislocations are observed in a higher magnification, as shown in Figure 8b, in addition to dislocation tangles near the grain boundary. It was reported that the grain refinement process of Ti alloys during ECAP could be attributed to the continuous dynamic recrystallization (CDRX) [43,45]. The process is often identified as follows: increasing dislocation density → dislocation tangles → sub-grains → LAGBs → CDRX grains. However, for the annealing behavior, the variation in microstructure and mechanical properties of the UFG TiFeB alloy is caused by recovery and SRX. In addition, it has been confirmed that the mechanisms of static recrystallization nucleation are generally divided into boundary bulge nucleation and sub-grains nucleation [46].

Figure 8c,d shows the TEM micrographs of the UFG TiFeB alloy sample annealed at 450 °C. Sub-grain boundaries become blurred with the appearance of HAGBs. Due to the low temperature, with the consumption of the strain hardened microstructure, static recovery (SRV) takes place, which is closely related to the recrystallization nuclei. The presence of SRV is responsible for the similar tensile properties between the UFG TiFeB alloy annealed at 450℃ and that annealed at 0℃.

As Figure 8e,f show, RGs and low dislocation density can be seen in the microstructure of the sample annealed at 650 °C. During the annealing process, sub-grains will disentangle, move and merge to adjacent sub-grains, which causes the integration of grain boundaries and the generation of HAGBs. This promotes the recrystallization nucleation. Therefore, the recrystallized nucleation is determined by sub-grain merging nucleation for the UFG TiFeB alloy annealed at 650 °C.

### 4.2. Effect of TiB Needles

**Figure 9:**
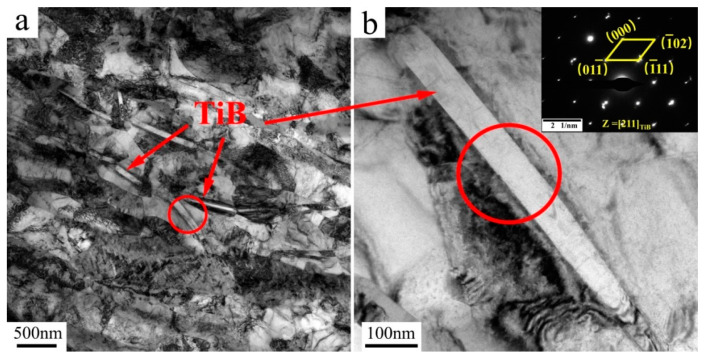
TEM images and SAED pattern of the TiB phase within the TiFeB alloy annealed at 450 °C for 1 h. (**a**) TEM images of the TiB phase; (**b**) SAED pattern of the circled TiB phase.

The TEM images of the TiB needles were detected for the UFG TiFeB alloy after annealing at 450 °C for 1 h, which are shown in Figure 9 with selected-area electron diffraction pattern (SAED). The circled part in Figure 9a is the needle-like TiB phase, and the circled part in Figure 9b is an enlarged view of the circled part in Figure 9a. As we all know, B does not dissolve in both α and β lattices in a titanium alloy; all the B added is utilized for the TiB needle formation, which, present at the grain boundaries, restrict their mobility at high temperatures due to the Zener drag mechanism [21]. 

### 4.3. Segregation of Fe

**Figure 10:**
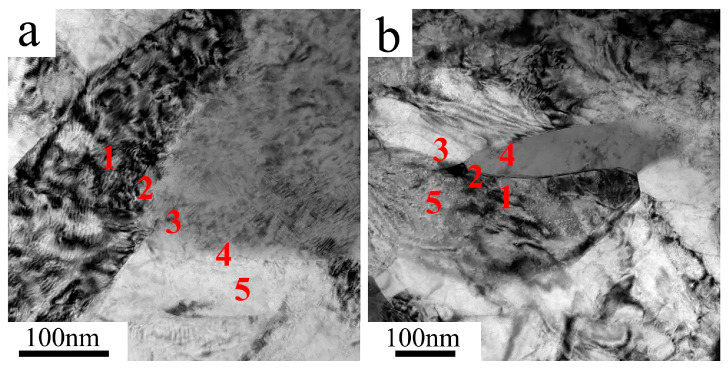
EDS spot measurements showing the chemical compositions of (**a**) the UFG alloy and (**b**) the alloy annealed at 450 °C for 1 h.

As an alloying element, the distribution of Fe in the microstructure of the UFG TiFeB alloy and the alloy annealed at 450 °C for 1 h were measured by the TEM-EDS tests (Figure 10). Several spots were selected and marked in the TEM micrographs with the numbers of 1, 2, 3, 4 and 5, which represent the distribution of Fe at the grain boundary, the α phase and the β phase. The chemical compositions (wt.%) of spots 1–5 under different conditions are listed in Table 1. As a strong β stable element, the solubility of Fe in the α phase is extremely limited; therefore, little Fe is detected in spots 1 and 5. On the contrary, there are larger amounts of Fe distributed on the β phase (spot 3 in Figure 10a), as well at the α/β grain boundaries (spots 2 and 4 in Figure 10a), both of which reach about 13%. After annealing at 450 °C, more Fe is detected at the grain boundaries and the mass fraction increases to 19.82% (spot 2 in Figure 10b), which is much higher than that of other areas.

Obviously, Fe is capable of diffusing to grain boundaries rapidly when the AT increases to 450 °C, which improves the thermal stability of the TiFeB alloy by reducing the grain boundary energy and inhibiting the mobility of grain boundaries. After ECAP, the microstructure of the UFG TiFeB alloy is mainly composed of dislocation cells, without obvious grain/subgrain boundaries during this period. When heat treatment is performed under a lower temperature, the sub-grain boundaries are filled in and an abundance of dislocations start to form and become characterized by LAGBs. Accompanying the increase in temperature, HAGBs form and their content increases gradually. At the same time, the Fe atoms are enriched at the grain boundaries, which inhibits the grain growth determined by the thermodynamical and kinetical aspect simultaneously.

In terms of thermodynamics, the alloying element Fe concentrates at the grain boundaries in the form of a solid solution or impurity atoms, leading to the “solute drag effect” [7] on grain boundaries and reducing the grain boundary energy. During this stage, due to the grain boundaries in thermodynamic equilibrium, the driving force of grain growth decreases and the grains cannot coarsen significantly before the solid solution atoms are dissolved and precipitated. While from the dynamics perspective, the stacking of Fe at the grain boundaries produces a “pinning effect” [47] on the grain boundaries, which hinders the movement of the grain boundaries and reduces their mobility. Therefore, the thermal stability of the UFG TiFeB alloy is improved.

### 4.4. Thermal Stability

**Figure 11:**
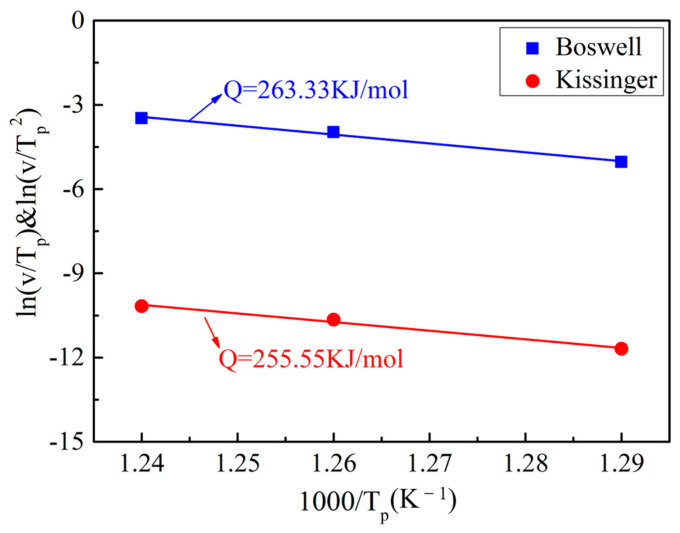
Recrystallization activation energy of the UFG TiFeB alloy determined by DSC.

The methods of Kissinger [48,49] and Boswell [50] were used to evaluate the activation energy of the recrystallization process and further illustrate the thermal stability of the UFG Ti-2Fe-0.1B alloy. The application of both methods is related to the change in the peak temperature in the DSC curves (Figure 11) at different heating rates according to the following expressions:

(1) $$\ln(v/T_{p}^{2})\;=\;C\;-\;(Q/RT_{p})\;\mathrm{Kissinger}\;\mathrm{equation},$$

*ln(v/T_p_)* = *C* − *(Q/RT_p_)* Boswell equation, (2)

where ν is the heating rate, *R* is the material constant (8.314 J mol^−1^K^−1^), *T_p_* is the peak temperature, *Q* is the recrystallization activation energy and C is a constant.

The activation energy is determined by the slope of the straight lines, which is obtained by fitting all of the data, as displayed in Figure 11. The results indicate that the recrystallization activation energies *Q* of the UFG TiFeB alloy obtained by the Kissinger and Boswell methods are 255.55 KJ/mol and 263.33 KJ/mol, respectively. In addition, the average recrystallization activation energy is 259.44 KJ/mol, which is much higher than the lattice self-diffusion activation energy of pure titanium of 169.1 KJ/mol [49]. According to previous reports, the activation energy of pure titanium after cold deformation is about 156.76 KJ/mol [13], while that of UFG CP titanium after the ECAP 8P process is 179 KJ/mol [51]. In contrast, the UFG TiFeB alloy has a higher recrystallization activation energy than UFG CP Ti, which means that the thermal stability of the UFG TiFeB alloy can rival that of UFG CP Ti, which was also found to be thermally stable up to 450 °C.

## 5. Conclusions

In the present work, the annealing behavior and thermal stability of the new UFG TiFeB alloy processed by ECAP were investigated. The main conclusions are summarized as follows:

1. Compared to the as-received UFG state, the microhardness of the sample remains almost unchanged and is kept stable at 242 HV when annealed at 450 °C during a long holding time. A good combination of strength (~786 MPa) and ductility (~16%) can be achieved for the UFG TiFeB alloy after annealing at 450 °C for 60 min.
2. When 300 °C < AT ≤ 450 °C, the average grain size still maintains an ultrafine level (0.91–1.03 μm), indicating that the UFG TiFeB alloy has a good thermal stability below 450 °C. Combined with the DSC test, the UFG TiFeB alloy has a higher recrystallization activation energy with an average value of 259.44 KJ/mol.
3. The Fe enriched at the grain boundaries of the UFG TiFeB alloy was detected after annealing at 450 °C. It was supposed to reduce the stored energy of the materials and the driving force of grain growth, which is beneficial to the thermal stability of the alloy. In addition, the pinning of a small amount of the TiB phase at the grain boundary also contributes to the thermal stability of the alloy.

## Figures and Tables

**Table 1:** Chemical compositions (wt.%) at spots 1–5 according to the EDS spot measurements.

Spots	UFG		450 °C/1 h	
	Ti	Fe	Ti	Fe
1	99.27	0.72	98.74	1.25
2	86.51	13.48	80.14	19.82
3	86.24	13.75	92.24	7.75
4	86.55	13.44	82.23	17.76
5	99.73	0.26	99.11	0.88

## Data Availability

Not Applicable.
